# Drastically magnetically tuned coupling strength and nonlinearity in CrSBr exciton-polaritons

**DOI:** 10.1038/s41377-026-02371-w

**Published:** 2026-06-26

**Authors:** Chun Li, Chao Shen, Xuekai Ma, Kwok Kwan Tang, Yutong Zhang, Nai Jiang, Xinyi Deng, Qing Wan, Jiepeng Song, Jiaqi Guo, Tian Lan, Hailong Fu, Feng Li, Yilin Wang, Xinfeng Liu, Qing Zhang

**Affiliations:** 1 School of Materials Science and Engineering, Peking University, Beijing, China; 2 State Key Laboratory of Semiconductor Physics and Chip Technologies, Institute of Semiconductors, Chinese Academy of Sciences, Beijing, China; 3 Center of Materials Science and Optoelectronics Engineering, University of Chinese Academy of Sciences, Beijing, China; 4 Department of Physics and Center for Optoelectronics and Photonics Paderborn (CeOPP), Paderborn University, Paderborn, Germany; 5 CAS Key Laboratory of Standardization and Measurement for Nanotechnology, CAS Center of Excellence for Nanoscience, National Center for Nanoscience and Technology, Beijing, China; 6 School of Integrated Circuits, Shandong Technology Center of Nanodevices and Integration, State Key Laboratory of Crystal Materials, Shandong University, Jinan, China; 7 School of Physics, Zhejiang University, Hangzhou, China; 8 Key Laboratory for Physical Electronics and Devices of the Ministry of Education & Shaanxi Key Laboratory of Information Photonic Technique, School of Electronic Science and Engineering, Faculty of Electronic and Information Engineering, Xi’an Jiaotong University, Xi’an, China

**Keywords:** Polaritons, Optical materials and structures, Polaritons

## Abstract

Two-dimensional van der Waals magnetic semiconductor CrSBr offers an ideal platform to achieve exciton-polaritons correlated with magnetic order for developing solid-state quantum, spintronic, and photonic devices. However, for the exciton-polaritons formed by lower-energy excitons (X_L_ ≈ 1.37 eV), the coupling strength and nonlinear optical response are almost inert to the external magnetic field. Here, we demonstrate robust strong coupling between higher-energy excitons (X_H_ ≈ 1.8 eV) and photons that persists up to room temperature, along with giant magnetic-field tunability. The Rabi splitting energy is tuned up to 100 meV within a moderate 0.45 T in-plane magnetic field due to changes in excitonic states during the spin transitions. Besides, we observe significantly enhanced polariton nonlinearity in the intermediate magnetic phase, which exhibits a distinct mode-number dependence and originates from magnon-assisted long-range attractive interactions and coupling strength reduction. These results advance the development of on-demand polariton platforms for spin-correlated quantum optoelectronics.

## Abstract (web summary)

An exciton-polariton in the 2D magnet CrSBr has a Rabi splitting energy and a nonlinear response strongly tunable by an external magnetic field.

## Graphical abstract

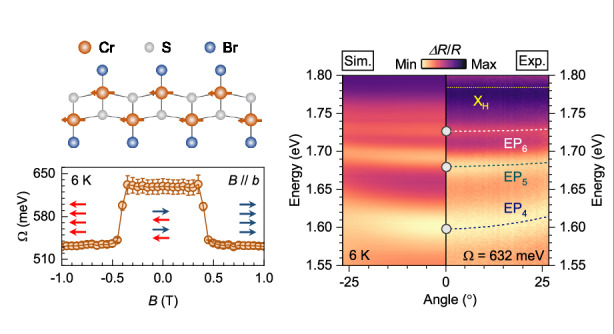

## Introduction

In semiconductor material systems, strong exciton-photon coupling gives rise to hybrid exciton-polaritons when the coupling strength exceeds the decay rates of both excitons and photons^1–3^. The half-light, half-matter nature of exciton-polaritons enables combining the advantages of both photons and excitons, sparking considerable interest in exploring exotic physics and designing advanced optoelectronic devices. For example, the light effective mass of the photonic component allows nonequilibrium polariton condensation at high temperatures and underpins pioneering studies of superfluidity, supersolidity, and dissipation-driven phase transition^4–8^. The strong interactions inherited from the excitonic component give rise to pronounced nonlinear optical response, paving new avenues for developing energy-efficient all-optical switches and quantum simulators^9–12^. Manipulating key parameters of exciton-polaritons, such as the coupling strength and nonlinearity, is therefore crucial for accessing emergent topological phases and quantum critical behaviors and actively controlling the performance of polaritonic devices^13,14^. Variations in the coupling strength fundamentally modify the physical picture of polaritons (e.g., their energy, effective mass, and scattering/relaxation rates)^15–17^. Likewise, tunable nonlinear optical response is particularly compelling to accommodate a wide range of scenarios, from strong interactions required for parametric scattering and spatiotemporal continuum generation to weak ones relevant for energy transfer and polaritonic chemistry^18–21^.

**Fig. 1:**
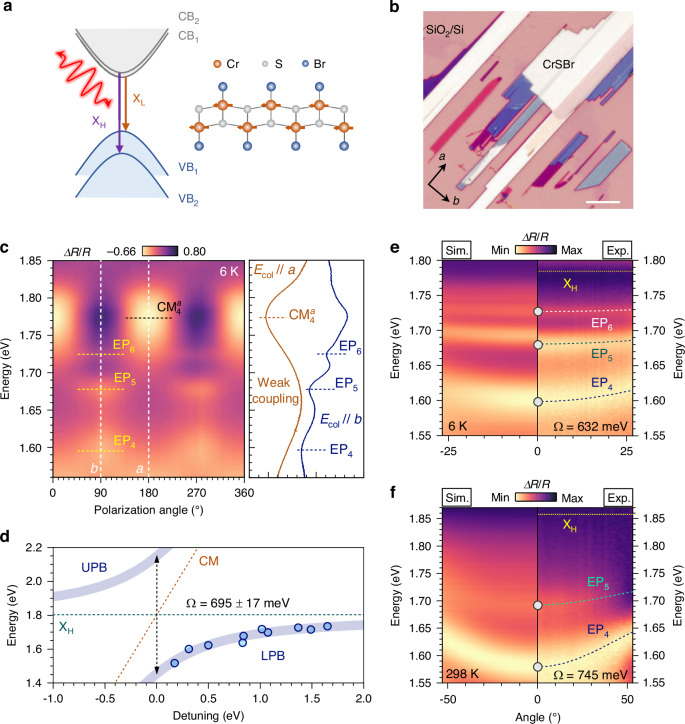
Strong coupling of X_H_ excitons and photons in CrSBr crystals. **a** Schematic of direct exciton transitions (X_H_ and X_L_) in CrSBr. CB and VB represent the conduction band and valence band, respectively. **b** Optical image of exfoliated CrSBr crystals on SiO_2_/Si. Black arrows represent the *a*-axis and *b*-axis. Scale bar is 5 μm. **c** Left panel: differential reflectance spectra as a function of the reflected-light polarization of the 354 nm-thick CrSBr crystal at 6 K. White dashed lines represent polarization along the *a*-axis and *b*-axis. Right panel: detailed reflectance spectra polarized along the *b*-axis (navy, strong coupling) and *a*-axis (brown, weak coupling). **d** Energy-detuning dispersion of CrSBr crystals with different thicknesses. The detuning energy is equal to the cavity mode energy minus the X_H_ exciton energy. Dots: experimental data at normal incidence. Navy curves: fitting results using the coupled harmonic oscillator model, giving an average Rabi splitting energy of 695 meV. UPB (LPB) stands for the upper (lower) polariton branch. **e**, **f** Simulated (left panel) and experimental (right panel) angle-resolved reflectance spectra of the 354 nm-thick CrSBr crystal at 6 K (**e**) and 298 K (**f**). Dotted curves and dashed curves denote the fitted X_H_ exciton and lower exciton-polariton branches using the coupled oscillator model. EP and CM represent the exciton-polariton and cavity mode, and subscripts (superscripts) indicate the mode number (polarization direction)

Magnetic fields enable real-time, non-invasive tuning of exciton-polariton properties, including energy dispersion, light–matter coupling strength, and nonlinearity^22^. Moreover, the interaction between exciton-polaritons and magnons facilitates the transfer of magnonic and spintronic information through polariton states, offering distinct advantages for optical detection and information transmission applications^23,24^. To meet this demand, magnetic van der Waals (vdW) semiconductors have attracted widespread interest as a bridge connecting strong light–matter interaction and magnetic excitations^25–27^. The vdW antiferromagnet NiPS_3_ exhibits strong coupling between magnetic excitons and photons, yet the Rabi splitting energy is quite small, measuring merely a few meV^28^. While pioneering works have achieved strong coupling of photons and lower-energy excitons (X_L_, ~1.37 eV, the first-band direct transition at the Γ point, see Fig. 1a) in CrSBr, the magnetic field tuning capability in coupling strength and nonlinearity is limited^29–31^. The higher-energy exciton (X_H_, ~1.8 eV) in CrSBr, arising from the second-band direct transition at the Γ point^32^, shows stronger magneto-exciton coupling due to enhanced delocalization^33–37^. This characteristic provides a promising platform for investigating magnetic tuning of exciton-polariton coupling strength and nonlinearity, though these phenomena remain unexplored to date.

Here, we report the observation of strong magnetic field engineered self-hybridized exciton-polaritons formed by strong coupling between X_H_ excitons and photons in CrSBr crystals. We find a remarkably large tuning of the Rabi splitting energy (up to 100 meV) during the antiferromagnetic (AFM) to ferromagnetic (FM) order change. Temperature can mediate the coupled exciton-polariton behaviors by manipulating the interactions between excitons and magnons/phonons. Further, the polariton nonlinearity is stronger in the intermediate magnetic (IM) phase than in the AFM and FM phases, indicating that spin order disruption enhances the attractive exciton-exciton interactions and coupling strength reduction assisted by magnons. These results pave the way for achieving controllable strong coupling in polaritonic optoelectronics and quantum applications.

## Results

Mesoscopic CrSBr crystals, with thicknesses ranging from tens to hundreds of nanometers, were prepared using mechanical exfoliation and transferred onto the SiO_2_/Si substrate. The elongated morphology of the crystals reflects the crystallographic anisotropy (Fig. 1b). The sufficient refractive index contrast at the top CrSBr/air and bottom CrSBr/SiO_2_ interfaces enables relatively high reflectivity, so the CrSBr crystal can act as a cavity to support vertical Fabry–Pérot resonance with cavity mode energy tuned by crystal thickness and thus self-hybridization in our experiments. The demonstration of self-hybridized exciton-polaritons relaxes the high-precision fabrication requirements for cavities and enables more dynamic tuning approaches, such as electric field, interface, and pressure^38^. To probe the excitonic resonance, two types of optical contrasts from differential reflectance spectroscopy (excited by a broad-band tungsten-halogen lamp, 360–2600 nm) and magnetic circular dichroism (MCD) spectroscopy (excited by a wavelength-tunable pulsed laser, 650–850 nm) were investigated (Methods). Polarization-resolved differential reflectance spectra at normal incidence of the 354 nm-thick CrSBr crystal indicate a reflectance dip at 1.764 eV when the polarization of the reflected light is along the *a*-axis, assigned to an uncoupled cavity mode (Fig. 1c). Due to the anisotropic refractive index, the corresponding uncoupled cavity mode along the *b*-axis is located at ~2.11 eV with a mode number *m* = 4 (~2.64 eV for *m* = 5 and ~3.17 eV for *m* = 6). In contrast, when the polarization is along the *b*-axis, a series of reflectance dips below 1.8 eV varying with the crystal thickness exist (Supplementary Fig. S1), which may be due to exciton-polaritons with different *m*. The calculated refractive index along the *b*-axis, according to the reflectance dip energy, sharply increases as energy approaches 1.8 eV (Supplementary Fig. S2). This obvious increase exceeds the refractive index variation described by the Sellmeier equation, extracted from the data polarized along the *a*-axis (without exciton effect), indicating the occurrence of strong exciton-photon coupling. The fitted X_H_ exciton oscillator strength using the Lorentz model for the dielectric function, considering the magnon effect (Supplementary Note S1), reaches 7.28 (eV)^2^, which is around four times greater than that of the X_L_ exciton^29–31^. The larger oscillator strength of X_H_ excitons can also be identified by fitting differential reflectance spectra (Supplementary Fig. S3), which is due to the larger exciton energy^2^. We also identify multiple exciton peaks above the X_H_ and X_L_ exciton energy (Supplementary Fig. S4), which may be due to indirect optical transitions with non-zero momentum near the Γ point^37^. However, these indirect excitons are unlikely to form exciton-polaritons due to their weak optical activity and small oscillator strength. Additionally, we used a coupled oscillator model to quantitatively fit the energy-detuning (Fig. 1d) and energy-wavevector dispersions (Supplementary Fig. S5) extracted from the thickness dependence of differential reflectance spectra, which show anti-crossing features. The fitted average Rabi splitting energy reaches 695 meV, much larger than the average dissipation energy of excitons (~50 meV) and photons (~50 meV), further confirming the formation of exciton-polaritons. Notably, the upper polariton branch is not observed in our experiments, likely attributed to strong continuum absorption above the X_H_ exciton energy^39^. Two representative exciton-polariton signals at 1.654 eV and 1.593 eV can be identified in both the differential reflectance and MCD spectra at 80 K, providing a consistency check (Supplementary Fig. S6). The polarization nature of the uncoupled cavity mode and coupled exciton-polaritons is independent of temperature and magnetic field (Supplementary Figs. S6, S7), consistent with the reported one-dimensional X_H_ exciton behavior^33,36^.

Figures 1e and 1f present angle-resolved reflectance spectra for a 354 nm-thick CrSBr crystal taken at 6 K and 298 K. The angle-resolved spectrum at 6 K exhibits three curved dispersions, well fitted using an exciton-polariton model with a Rabi splitting energy of 632 meV. This strong coupling is robust across crystals with similar dimensions (Supplementary Fig. S8), because they possess comparable excitonic and photonic properties to form self-hybridized exciton-polaritons, highlighting the reliability of CrSBr as a material platform for exciton-polariton applications free from the complicated cavity design. The normalized coupling strength of 0.177 reveals that the exciton-photon coupling may have entered the ultrastrong coupling regime. To explore it, we used the ultrastrong coupling model with non-negligible higher-order effects and estimated a Rabi splitting energy of ~800 meV (Supplementary Fig. S9 and Note S2), which is close to the value extracted by the strong coupling model. Nevertheless, we have not yet directly observed phenomena typically associated with the ultrastrong coupling regime, such as correlated photon-pair generation and vacuum Bloch–Siegert shift^40,41^. Thus, we adopt the strong coupling model in the following discussion, while further studies to unambiguously verify ultrastrong coupling will be carried out in future work. The larger dispersion curvature and increased linewidth in the lower branches indicate a reduction in effective mass and an increase in dissipation with the mixing of more photons. Due to phonon-assisted exciton linewidth broadening, only two exciton-polariton branches are observed at 298 K, with a fitted Rabi splitting energy of 745 meV. Further, the simulated angle-resolved reflectance spectra using the finite-difference time-domain (FDTD) method are closely aligned with experimental observations.

**Fig. 2:**
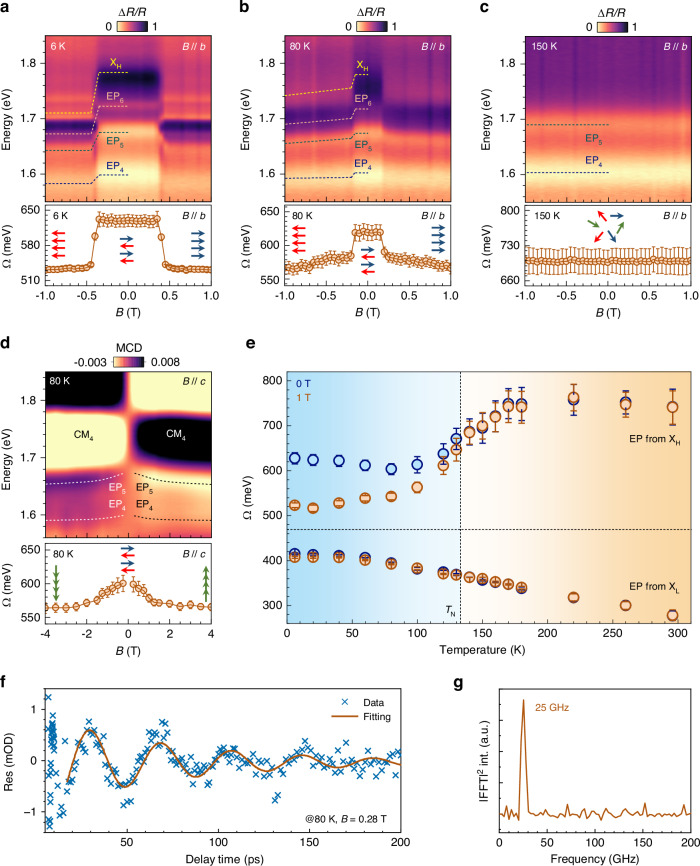
Magnetic tuning of Rabi splitting energy. **a–d** Upper panel: magnetic field dependence of normalized differential reflectance spectra at 6 K (**a**), 80 K (**b**), 150 K (**c**), and MCD spectra at 80 K (**d**) of the 354 nm-thick CrSBr crystal. Lower panel: Magnetic field dependence of fitted Rabi splitting energies. Colored arrows show the possible orientation of magnetization. **e** Temperature dependence of the Rabi splitting energy under *B* = 0 T (navy) and *B* = 1 T (brown). The data above (below) the horizontal dashed line correspond to exciton-polaritons formed by X_H_ excitons (X_L_ excitons). The vertical dashed line represents the Néel temperature *T*_N_. **f** Oscillatory components probed at X_H_ exciton-polariton energy in the transient reflectance spectra at 80 K and under *B* = 0.28 T, where the residual parts are extracted by subtracting the decay background. **g** Fast Fourier transform of the data in (**f**), indicating a peak frequency of ~25 GHz, consistent with the magnon frequency

The behaviors of X_H_ excitons and the corresponding exciton-polaritons are highly dependent on the magnetic order and can be effectively tailored by an applied external magnetic field. At 6 K, reflectance spectra at normal incidence as a function of the in-plane magnetic field (*B*, parallel to the easy *b*-axis) show that all exciton-polariton branches maintain the constant energies when *B* is less than 0.35 T, followed by an abrupt redshift between 0.35–0.45 T, after which the energies stabilize again as *B* exceeds 0.45 T (Fig. 2a and Supplementary Fig. S10). This sudden redshift arises from changes in interlayer electronic hybridization due to spin flipping, accomplished by the transition from AFM across IM to FM order^26^. Interestingly, the Rabi splitting energy also sharply decreases from 632 meV to 535 meV across the spin flipping transition. In contrast, the Rabi splitting energy for the coupling of X_L_ excitons and photons is almost independent of the magnetic order (Supplementary Fig. S11). Therefore, the resulting modulation of light velocity and effective mass of exciton-polaritons, 100 meV below the X_H_ exciton energy, respectively reaches 10.3% and 26.1%, 1–2 orders of magnitude higher than those of X_L_ exciton (Supplementary Fig. S12 and Note S3). The giant magnetic modulation of the Rabi splitting energy is due to the fact that the wavefunction of X_H_ excitons exhibits a larger inter-site character, involving interactions between multiple Cr atomic sites in space, where electrons and holes may be located on different Cr atoms^33,36^. Therefore, X_H_ excitons are more sensitive to changes in interlayer interactions between Cr atoms during the spin flipping, leading to the exciton wavefunction delocalization and the reduction of the Rabi splitting energy. In contrast, the contribution of local Cr atomic sites plays a crucial role in the wavefunction of X_L_ excitons, resulting in negligible wavefunction delocalization and thus Rabi splitting energy tuning by magnetic fields. Further, the fitted energy redshift of X_H_ excitons is ~78 meV (Supplementary Fig. S10), nearly five times that of X_L_ excitons (~16 meV, Supplementary Fig. S11), reflecting a dramatic field-induced band structure change associated with the X_H_ exciton transition. The global vacuum energy (the sum of vacuum photon energy and exciton energy^42^) in the ultrastrong coupling system undergoes a variation (depending on coupling strength) compared with the uncoupled system, so that the directly measured energy shift of X_H_ excitons in a few-layer sample without exciton-polaritons, ~88 meV (Supplementary Fig. S13), exhibits a slight difference from the fitted value (~78 meV) using the strong coupling model. When the temperature increases to 80 K, similar switching behaviors in Rabi splitting energy, X_H_ exciton-polariton energy, and X_H_ exciton energy are observed (Fig. 2b and Supplementary Fig. S10). The critical magnetic field for the spin flipping transition is lower (~0.15–0.20 T), above which a slight energy redshift still occurs. These results are due to thermal fluctuations weakening the pristine AFM coupling and field-induced FM coupling^31^. At 150 K (above the Néel temperature of 132 K for bulk CrSBr), CrSBr transforms into a paramagnetic state, and the magnetic response of both X_H_ excitons and the corresponding exciton-polaritons vanishes completely (Fig. 2c and Supplementary Fig. S10).

MCD spectroscopy was employed to further verify the magnetic tuning of strong coupling. Figure 2d presents the out-of-plane magnetic field (parallel to the hard *c*-axis) dependence of MCD spectra at 80 K. The exciton-polariton branches exhibit a continuous redshift with increasing magnetic field, consistent with the spin canting process. The redshift rate slows down significantly at *B* > 1 T, accompanied by saturation of the MCD intensity growth (Supplementary Fig. S14), highlighting the critical magnetic field for the AFM–FM phase transition. Similarly, fitted Rabi splitting energy and X_H_ exciton energy also continuously undergo a reduction and gradually stabilize beyond *B* = 1 T (Fig. 2d and Supplementary Fig. S10). The consistency between the reflectance and MCD spectra at the same temperature of 80 K, across both AFM and FM phases, verifies the reliability of the data.

The temperature dependence of reflectance spectra was collected to unlock the evolution of exciton-polaritons influenced by excitons interacting with incoherent magnons and phonons (Supplementary Fig. S15). At *B* = 0 T, the Rabi splitting energy monotonically decreases below 100 K, and then increases between 100 and 200 K. At *B* = 1 T (above the critical field for the AFM–FM transition), an accelerated increase occurs below 200 K, resulting in a continuous reduction of field-induced tuning of Rabi splitting energy from ~100 meV (~16%) at 6 K to around zero above 140 K (Fig. 2e). Specifically, the temperature-dependent behaviors of the Rabi splitting energy closely mirror the polariton energy and the fitted X_H_ exciton energy, all of which can be well described by the exciton-magnon coupling and exciton-phonon coupling theory (Supplementary Fig. S16, Notes S4 and S5), suggesting exciton-photon coupling strongly correlated with spin and phonon. Proof of exciton-magnon coupling is provided by transient reflectance measurements, where we observe an oscillatory signal after subtracting the incoherent decay background (Fig. 2f, probed at the X_H_ exciton-polariton energy). By performing a fast Fourier transform of the oscillatory response, we obtain a peak frequency of ~25 GHz (Fig. 2g), consistent with the reported magnon frequency in CrSBr^27^. The blueshift of the X_H_ exciton energy below 200 K may be due to a positive temperature slope of the gap driven by the thermal expansion term^43^. There are still several meV magnetic tuning amounts above 140 K, possibly due to the existing short-range correlations above the Néel temperature^29^. As a comparison, the Rabi splitting energy of exciton-polaritons formed by X_L_ excitons can hardly be tuned by an external magnetic field.

**Fig. 3:**
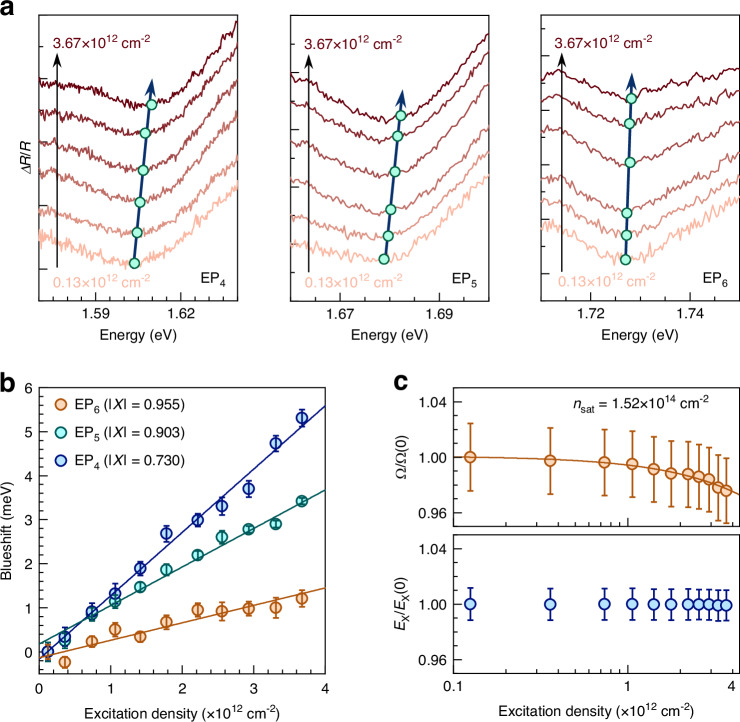
X_H_ exciton-polariton nonlinearity. **a**, **b** Excitation density dependence of reflectance spectra (**a**) and blueshift (**b**) for three exciton-polariton branches in the 354 nm-thick CrSBr crystal excited by a broad-band pulsed laser spectrally filtered from a supercontinuum laser. The central energies of exciton-polaritons are marked with cyan circles in (**a**). |*X*| is exciton fraction. **c** Fitted Rabi splitting energy (upper panel) and X_H_ exciton energy (lower panel) relative to the Rabi splitting energy and X_H_ exciton energy at the lowest excitation density as a function of excitation density according to (**a**). The solid curve is the fitting result using the model in Supplementary Note S6

Probing and manipulating polariton nonlinearity are crucial in accessing many-body quantum phenomena and quantum optical devices. We characterized the nonlinear response of exciton-polaritons by performing excitation density-dependent reflectance measurements, where the excitation source was derived from a supercontinuum laser spectrally filtered from 695–810 nm (Methods). Figure 3a shows the representative reflectance spectra at normal incidence of three exciton-polariton branches in the 354 nm-thick crystal, and the polariton energy is obtained by fitting the reflectance dips using a Lorentzian function (Supplementary Fig. S17). As the excitation density increases, all branches exhibit a linear blueshift, and the magnitude of the blueshift gradually increases as the exciton component decreases (Fig. 3b). Within the excitation density range of interest, the fitted X_H_ exciton energy remains almost unchanged, reflecting the suppressed long-range excitonic interactions (Fig. 3c). Similar behavior has been reported in other zero-dimensional and one-dimensional exciton systems with highly confined wavefunctions^18,28^. We also examine the scaling of polariton interaction strength versus exciton fraction (Supplementary Fig. S18). The saturation term due to phase-space filling is stronger than the interaction term due to exciton-exciton Coulomb interaction by nearly an order of magnitude, further proving that phase-space filling plays a dominant role in the polariton nonlinearity, different from the leading exciton-exciton interaction in exciton nonlinearity^33^. We can exclude the possibility of laser-induced heating, as it would cause a redshift of the X_H_ exciton and exciton-polariton energies according to our temperature-dependent measurements (Supplementary Figs. 15, 16), which contradicts the observed behaviors. The cavity mode energy renormalization based on the dispersion effect has also been considered to induce polariton nonlinearity. Here, following previous studies on the nonlinearity of 2D semiconductors^18^, we assume that the cavity mode energy does not change with excitation density, partly because the uncoupled X_H_ exciton peak is invisible and thus may not significantly modify the background refractive index around the resonance energy^44^. Nevertheless, since we cannot directly address the cavity modes along the *b*-axis from the experiments, complete exclusion still requires further research. Due to the Pauli exclusion principle (phase-space filling effect), the Rabi splitting energy saturates as the excitation density approaches the fitted saturation exciton density *n*_s_ of 1.52 × 10^14 ^cm^−2^ (Supplementary Note S6). Considering the anisotropic dielectric function (*ε*_b_/*ε*_a_ = 3.75) and the elliptical distribution of the exciton wavefunction^29,45^, the X_H_ exciton Bohr radius along the *b*-axis (*a*_b_) and *a*-axis (*a*_a_) can be estimated according to ${a}_{{\rm{a}}}\times {a}_{{\rm{b}}}={a}_{{\rm{a}}}\times 3.75{a}_{{\rm{a}}}=0.07/{n}_{{\rm{s}}}$, giving *a*_b_ = 0.90 nm and *a*_a_ = 0.25 nm. The consistency of these results indicates that the strongly localized nature of X_H_ excitons leads to the extremely large exciton oscillator strength and thus the occurrence of strong coupling.

**Fig. 4:**
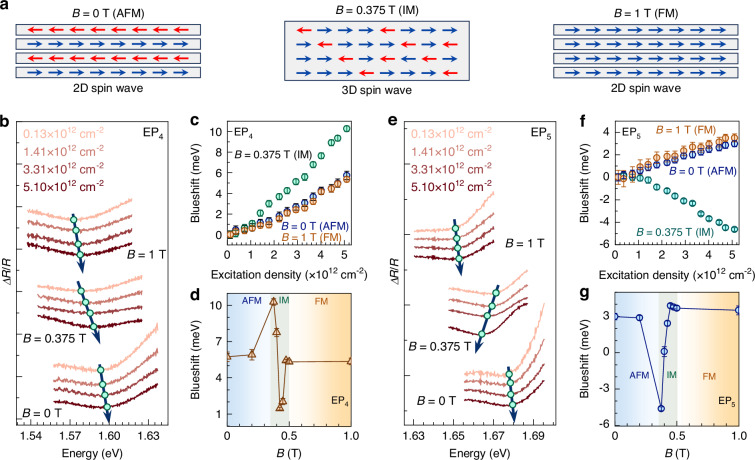
Magnetic tuning of polariton nonlinearity. **a** Schematic of spin ordering disruption resulting in the transformation of the spin wave from 2D to 3D. **b**, **c**, **e**, **f** Excitation density dependence of reflectance spectra (**b**, **e**) and blueshift (**c**, **f**) for *m* = 4 (**b**, **c**) and *m* = 5 polariton branches (**e**, **f**) in the 354 nm-thick CrSBr crystal under *B* = 0 T (AFM phase), 0.375 T (IM phase), and 1 T (FM phase). The central energies of exciton-polaritons are marked with cyan circles in (**b**) and (**e**). **d**, **g** Magnetic field dependence of blueshift at 5.1 × 10^12 ^cm^-2^ for *m* = 4 (**d**) and *m* = 5 branches (**g**). Negative blueshift indicates redshift

The effect of magnetic field (parallel to the easy *b*-axis) on polariton nonlinearity has been further studied. Figures 4b and 4e show the excitation density-dependent reflectance spectra for two exciton-polariton branches at *B* = 0 T (AFM phase), 0.375 T (IM phase), and 1 T (FM phase). Compared to *B* = 0 T, both branches at *B* = 1 T exhibit a similar blueshift. However, at *B* = 0.375 T, the blueshift at excitation density of 5.1 × 10^12 ^cm^−2^ of mode number *m* = 4 branch, increases to 10 meV, twice that at *B* = 0 T (Fig. 4c), and *m* = 5 branch exhibits a 5 meV redshift, in contrast to the 3 meV blueshift at *B* = 0 T (Fig. 4f). We attribute these results to additional long-range interaction mediated by magnons in the IM phase. The stronger exciton-magnon coupling enhances the attractive exciton-exciton interaction when the spin flipping begins^33^, resulting in an energy redshift of X_H_ excitons and *m* = 5 branch with a large exciton fraction. Meanwhile, the magnon-assisted long-range interactions lead to a decrease in exciton saturation density, which accelerates the Rabi splitting energy reduction and thereby the blueshift of *m* = 4 branch (Supplementary Note S7). Here, we do not present the magnetic response of polariton nonlinearity from other branches, because *m* = 6 branch cannot be detected in the IM phase, possibly due to overlap with the indirect exciton signal, while *m* < 4 branches are obscured by the signal from the X_L_ exciton-polaritons. Exciton-exciton interactions and phase-space filling are primarily responsible for the polariton nonlinearity in the conventional systems, requiring exciton wavefunctions to overlap and fill phase-space at high excitation densities. Due to the strongly localized exciton wavefunctions in CrSBr, these two channels are suppressed, as revealed by the unchanged exciton energy and weak Rabi splitting energy saturation. However, due to the strong coupling between excitons and magnons, as well as the spatial extension of spin waves, exciton-exciton interactions and phase-space filling can occur over long ranges via magnons as mediators at excitation density on the order of 10^12 ^cm^−2^, where the average exciton spacing is one order of magnitude higher than the Bohr radius. In the AFM and FM phases, spin waves mainly retain their in-plane (2D-like) characteristics (Fig. 4a) due to weak interlayer exchange interactions in the vdW magnet CrSBr^46^. In the IM phase, incomplete alignment of spins across different layers leads to enhanced interlayer coupling, which facilitates the formation of interlayer spin waves (3D-like) and thus both magnon-mediated long-range attractive interactions and the reduction of the coupling strength, consequently strongly affecting the polariton nonlinearity. An enhanced exciton-magnon coupling has been observed in the IM phase^27^, which supports our explanation.

We have added data at other magnetic fields and confirmed that the energy shift of the exciton-polaritons by varying excitation density can be continuously controlled in the IM phase (Figs. 4d, 4g). The shift of *m* = 4 (*m* = 5) branch can be tuned from a redshift of 5 meV to a blueshift of 4 meV (from a blueshift of 1 meV to 10 meV). Since spin flipping occurs rapidly within a small magnetic field range in our configuration, the polariton nonlinearity dramatically increases and then quickly returns, different from the gradually evolving exciton-exciton interaction observed during spin canting in a recent work^33^. The demonstrated sensitive and customizable polariton nonlinearity provides a promising approach for developing flexible nonlinear optical switches and quantum simulators.

## Discussion

Real-time, non-destructive tuning of the coupling strength is essential for the development of exciton-polariton applications with engineered optical and electrical properties. Precise control over the coupling strength enables manipulation of polariton scattering and relaxation^16^, coherence of coupled systems^47^, entanglement between quantum light sources^48^, and eigenstates of qubits^49^—capabilities that are critical for designing low-threshold polariton lasers, polariton gates, and neuromorphic computing. In this work, we present a suitable candidate to fulfill these requirements. We demonstrate strong coupling between magnetically dressed X_H_ excitons and photons in the vdW CrSBr crystals with a Rabi splitting energy up to 745 meV and operation temperature range from 6 to 298 K. Interestingly, we find that the Rabi splitting energy of exciton-polaritons formed by X_H_ excitons is extensively tuned by an applied in-plane magnetic field within 1 T, and the magnetic tunability is 1–2 orders of magnitude larger than that of previously studied CrSBr X_L_ excitons and/or other vdW semiconductor excitons, which is due to significant exciton wavefunction delocalization. More in-depth experiments are expected to be conducted in the next efforts to probe phenomena associated with the ultrastrong coupling regime, such as ground-state virtual photons and counter-rotating effect^40,50^.

In addition, we report a unique polariton nonlinearity, highlighting the profound influence of strong light–matter coupling on many-body interactions. Unlike exciton-polaritons generated in non-magnetic materials, changes in the magnetic order of CrSBr affect the magnon-assisted polariton attraction interaction and phase-space filling effect, leading to unprecedented magnetic-field-tunable nonlinearity. We expect to investigate the variations in exciton-magnon coupling using transient spectroscopy under continuously varying magnetic fields as a next step, which will facilitate the quantitative analysis of magnon-assisted polariton nonlinearity. On the other hand, unlike the conventional saturation nonlinearity of excitons with either redshift or blueshift^33^, X_H_ exciton-polaritons exhibit mode-dependent nonlinear responses with two-way shifts over a broader spectral range due to their hybrid nature, where the excitonic and photonic fractions govern their interaction strengths. This provides an additional degree of freedom in designing polaritonic devices—by selectively exciting specific modes via cavity engineering, we can tailor nonlinearities for wide applications^14^. The pump-induced blueshift observed in this work (up to 10 meV) is already comparable to values used for developing functional polaritonic switches^9^. The key challenge for future exploration lies in reducing the polariton linewidth to well below the blueshift magnitude—through coupling with a high-quality-factor cavity, resonant pumping, or realizing polariton condensation—to enhance the on/off ratio. We believe our findings provide a self-hybridized platform in simple and natural structures to explore underlying physics involving magnetically dressed exciton-polaritons. Precise control over the coupling between cavity photons and CrSBr excitons, e.g., introducing microcavities with finely tuned optical modes, is highly desirable for practical applications in the future.

## Materials and methods

### Sample preparations

The mesoscopic CrSBr crystals were prepared using mechanical exfoliation from the bulk crystals (purchased from Nanjing MKNANO Tech. Co., Ltd.) and transferred onto the pre-cleaned SiO_2_/Si substrate (SiO_2_ thickness: 285 nm).

### Steady-state reflectance measurements

The angle-resolved measurements at room temperature were carried out by using a home-built Fourier imaging setup, where the reflectance (source: tungsten-halogen lamp, 360–2600 nm, Thorlabs, SLS201L) signals were collected through a 50× objective (N.A. = 0.8), then sent to a spectrometer (Horiba, iHR320) after passing through a square aperture to control a collection area of ~25 μm^2^. Low-temperature measurements were carried out in a cryostat (Montana, CR-562), equipped with a 50× objective (N.A. = 0.45) for angle-resolved reflectance, and the spectra were detected using a spectrometer (Horiba, iHR550). For excitation density-dependent spectroscopy, the excitation source was derived from a supercontinuum laser (NKT Photonics, Fianium WhiteLase) with a repetition rate of 80 MHz and a pulse width of 6 ps, which was spectrally filtered from 695–810 nm using a band-pass filter with a controllable tilt angle. The spot size was focused to ~7 μm^2^ by a 50× objective (N.A. = 0.45), and the integrated pump power was increased from 0.16 to 4.59 mW. The reflected light was attenuated by optical density filters before entering the spectrometer to protect the charge-coupled device. The in-plane magnetic field-dependent measurements were realized using an electromagnet (Yingpu Magnetoelectric, WD-130).

### Magnetic circular dichroism measurements

In the magnetic circular dichroism (MCD) measurements, the excitation source was a wavelength-tunable laser (650–850 nm) derived from a supercontinuum laser (YSL Photonics, SC-pro) with a repetition rate of 80 MHz and a pulse width of 6 ps, equipped with a monochromator (Horiba, iHR320). The excitation light was focused to a spot area of ~10 μm^2^ by a 10× objective (N.A. = 0.25), and the pump power at each wavelength was ~0.3 mW. The detection of the reflected light from the sample was achieved using a Si photodetector. MCD signals were measured with two lock-in amplifiers, referencing a 177 Hz chopper and a 50 kHz photoelastic modulator. The samples were mounted inside a cryostat (Janis, ST-500) cooled by liquid nitrogen, and the out-of-plane magnetic field was provided by a superconducting magnet (CryoMagnetics).

### Transient reflectance measurements

Transient reflectance measurements were obtained by the HELIOS commercial fs-T system (Ultrafast Systems). The fundamental 800 nm pulsed light (1 kHz, 80 fs) from a Coherent Astrella regenerative amplifier was passed through a BBO crystal to generate a 400 nm pumping beam (power of 0.23 mW). A white light continuum probe beam ranging from 430 to 775 nm was produced by focusing a small part of the fundamental 800 nm beam onto a sapphire window. The waist radius of the pump beam and probe beam were ~40 μm and 2 μm, respectively. The samples were held at 80 K using a cryostat (Cryo Industry) cooled by liquid nitrogen, and the magnetic field of ~0.28 T along the *c*-axis was applied using a permanent magnet.

### FDTD simulations

FDTD simulations were performed by considering the refractive index in the Lorentz model. The lateral size of the CrSBr crystal was assumed to be infinite. The excitation source was a plane wave source, and a far-field monitor was utilized to collect the reflected signal at different angles.

## Supplementary information

Supplementary Information

## Data Availability

The data that support the findings of this study are available from the corresponding author upon reasonable request.

## References

[1] Bloch, J. et al. Strongly correlated electron–photon systems. *Nature* **606**, 41–48 (2022). 35614214 10.1038/s41586-022-04726-w

[2] Hopfield, J. J. Theory of the contribution of excitons to the complex dielectric constant of crystals. *Phys. Rev.* **112**, 1555–1567 (1958).

[3] Słowik, K. et al. Strong coupling of optical nanoantennas and atomic systems. *Phys. Rev. B* **88**, 195414 (2013).

[4] Deng, H. et al. Condensation of semiconductor microcavity exciton polaritons. *Science* **298**, 199–202 (2002). 12364801 10.1126/science.1074464

[5] Song, J. P. et al. Room-temperature continuous-wave pumped exciton polariton condensation in a perovskite microcavity. *Sci. Adv.* **11**, eadr1652 (2025). 39879295 10.1126/sciadv.adr1652 PMC11777180

[6] Lerario, G. et al. Room-temperature superfluidity in a polariton condensate. *Nat. Phys.* **13**, 837–841 (2017).

[7] Trypogeorgos, D. et al. Emerging supersolidity in photonic-crystal polariton condensates. *Nature* **639**, 337–341 (2025). 40044862 10.1038/s41586-025-08616-9

[8] Fontaine, Q. et al. Kardar–Parisi–Zhang universality in a one-dimensional polariton condensate. *Nature* **608**, 687–691 (2022). 36002483 10.1038/s41586-022-05001-8

[9] Zasedatelev, A. V. et al. Single-photon nonlinearity at room temperature. *Nature* **597**, 493–497 (2021). 34552252 10.1038/s41586-021-03866-9

[10] Li, H. et al. All-optical temporal logic gates in localized exciton polaritons. *Nat. Photonics* **18**, 864–869 (2024).

[11] Wu, X. X. et al. Exciton polariton condensation from bound states in the continuum at room temperature. *Nat. Commun.* **15**, 3345 (2024). 38637571 10.1038/s41467-024-47669-8 PMC11026397

[12] Sedov, E. & Kavokin, A. Polariton lattices as binarized neuromorphic networks. *Light Sci. Appl.* **14**, 52 (2025). 39819972 10.1038/s41377-024-01719-4 PMC11739516

[13] Zhao, H. F. et al. Sub-picosecond topological phase transition in nonlinear exciton–polariton superlattices. *Nat. Phys.* **21**, 1806–1812 (2025).

[14] Kavokin, A. et al. Polariton condensates for classical and quantum computing. *Nat. Rev. Phys.* **4**, 435–451 (2022).

[15] Gao, W. L. et al. Continuous transition between weak and ultrastrong coupling through exceptional points in carbon nanotube microcavity exciton–polaritons. *Nat. Photonics* **12**, 362–367 (2018).

[16] Le Roux, F. et al. Efficient anisotropic polariton lasing using molecular conformation and orientation in organic microcavities. *Adv. Funct. Mater.* **32**, 2209241 (2022).

[17] Wu, T. T. et al. Ultrastrong exciton-plasmon couplings in WS_2_ multilayers synthesized with a random multi-singular metasurface at room temperature. *Nat. Commun.* **15**, 3295 (2024). 38632230 10.1038/s41467-024-47610-z PMC11024105

[18] Zhang, L. et al. Van der Waals heterostructure polaritons with moiré-induced nonlinearity. *Nature* **591**, 61–65 (2021). 33658695 10.1038/s41586-021-03228-5

[19] Zhao, J. X. et al. Nonlinear polariton parametric emission in an atomically thin semiconductor based microcavity. *Nat. Nanotechnol.* **17**, 396–402 (2022). 35288672 10.1038/s41565-022-01073-9

[20] Walker, P. M. et al. Spatiotemporal continuum generation in polariton waveguides. *Light Sci. Appl.* **8**, 6 (2019). 30651981 10.1038/s41377-019-0120-7 PMC6333623

[21] Ebbesen, T. W., Rubio, A. & Scholes, G. D. Introduction: polaritonic chemistry. *Chem. Rev.* **123**, 12037–12038 (2023). 37936399 10.1021/acs.chemrev.3c00637

[22] Bloch, J., Carusotto, I. & Wouters, M. Non-equilibrium Bose–Einstein condensation in photonic systems. *Nat. Rev. Phys.* **4**, 470–488 (2022).

[23] Yuan, H. Y. et al. Quantum magnonics: when magnon spintronics meets quantum information science. *Phys. Rep.* **965**, 1–74 (2022).

[24] Pirro, P. et al. Advances in coherent magnonics. *Nat. Rev. Mater.* **6**, 1114–1135 (2021).

[25] Burch, K. S., Mandrus, D. & Park, J. G. Magnetism in two-dimensional van der Waals materials. *Nature* **563**, 47–52 (2018). 30382199 10.1038/s41586-018-0631-z

[26] Wilson, N. P. et al. Interlayer electronic coupling on demand in a 2D magnetic semiconductor. *Nat. Mater.* **20**, 1657–1662 (2021). 34312534 10.1038/s41563-021-01070-8

[27] Diederich, G. M. et al. Tunable interaction between excitons and hybridized magnons in a layered semiconductor. *Nat. Nanotechnol.* **18**, 23–28 (2023). 36577852 10.1038/s41565-022-01259-1

[28] Dirnberger, F. et al. Spin-correlated exciton–polaritons in a van der Waals magnet. *Nat. Nanotechnol.* **17**, 1060–1064 (2022). 36097046 10.1038/s41565-022-01204-2

[29] Dirnberger, F. et al. Magneto-optics in a van der Waals magnet tuned by self-hybridized polaritons. *Nature* **620**, 533–537 (2023). 37587298 10.1038/s41586-023-06275-2

[30] Wang, T. T. et al. Magnetically-dressed CrSBr exciton-polaritons in ultrastrong coupling regime. *Nat. Commun.* **14**, 5966 (2023). 37749106 10.1038/s41467-023-41688-7 PMC10520032

[31] Li, C. et al. 2D CrSBr enables magnetically controllable exciton-polaritons in an open cavity. *Adv. Funct. Mater.* **34**, 2411589 (2024).

[32] Shao, Y. M. et al. Magnetically confined surface and bulk excitons in a layered antiferromagnet. *Nat. Mater.* **24**, 391–398 (2025). 39972108 10.1038/s41563-025-02129-6

[33] Datta, B. et al. Magnon-mediated exciton–exciton interaction in a van der Waals antiferromagnet. *Nat. Mater.* **24**, 1027–1033 (2025). 40119034 10.1038/s41563-025-02183-0

[34] Nessi, L. et al. Magnetic field tunable polaritons in the ultrastrong coupling regime in CrSBr. *ACS. Nano* **18**, 34235–34243 (2024). 39639608 10.1021/acsnano.4c11799

[35] Komar, R. et al. Colossal magneto-excitonic effects in 2D van der Waals magnetic semiconductor CrSBr. Print at https://arxiv.org/abs/2409.00187 (2024).

[36] Shi, J. et al. Giant magneto-exciton coupling in 2D van der Waals CrSBr. *ACS. Nano* **19**, 29977–29987 (2025). 40802066 10.1021/acsnano.5c00407

[37] Śmiertka, M. et al. Distinct magneto-optical response of Frenkel and Wannier excitons in CrSBr. *Nat. Commun.* **17**, 1777 (2026). 41547858 10.1038/s41467-026-68482-5 PMC12916971

[38] Canales, A. et al. Abundance of cavity-free polaritonic states in resonant materials and nanostructures. *J. Chem. Phys.* **154**, 024701 (2021). 33445887 10.1063/5.0033352

[39] Guillet, T. & Brimont, C. Polariton condensates at room temperature. *Comptes Rendus. Phys.* **17**, 946–956 (2016).

[40] Liberato, S. D., Ciuti, C. & Carusotto, I. Quantum vacuum radiation spectra from a semiconductor microcavity with a time-modulated vacuum Rabi frequency. *Phys. Rev. Lett.* **98**, 103602 (2007). 17358533 10.1103/PhysRevLett.98.103602

[41] Li, X. W. et al. Vacuum Bloch–Siegert shift in Landau polaritons with ultra-high cooperativity. *Nat. Photonics* **12**, 324–329 (2018).

[42] Baranov, D. G. et al. Ultrastrong coupling between nanoparticle plasmons and cavity photons at ambient conditions. *Nat. Commun.* **11**, 2715 (2020). 32483151 10.1038/s41467-020-16524-x PMC7264206

[43] Fasahat, S. et al. Sign of the gap temperature dependence in CsPb(Br, Cl)_3_ nanocrystals determined by Cs-rattler-mediated electron–phonon coupling. *J. Phys. Chem. Lett.* **16**, 1134–1141 (2025). 39846450 10.1021/acs.jpclett.4c03491 PMC11915369

[44] Yagafarov, T. et al. Mechanisms of blueshifts in organic polariton condensates. *Commun. Phys.* **3**, 18 (2020).

[45] Li, Q. Y. et al. Two-dimensional magnetic exciton polariton with strongly coupled atomic and photonic anisotropies. *Phys. Rev. Lett.* **133**, 266901 (2024). 39879023 10.1103/PhysRevLett.133.266901

[46] Bae, Y. J. et al. Exciton-coupled coherent magnons in a 2D semiconductor. *Nature* **609**, 282–286 (2022). 36071189 10.1038/s41586-022-05024-1

[47] Shi, L. et al. Spatial coherence properties of organic molecules coupled to plasmonic surface lattice resonances in the weak and strong coupling regimes. *Phys. Rev. Lett.* **112**, 153002 (2014). 24785036 10.1103/PhysRevLett.112.153002

[48] Dousse, A. et al. Ultrabright source of entangled photon pairs. *Nature* **466**, 217–220 (2010). 20613838 10.1038/nature09148

[49] Martins, F. et al. Noise suppression using symmetric exchange gates in spin qubits. *Phys. Rev. Lett.* **116**, 116801 (2016). 27035316 10.1103/PhysRevLett.116.116801

[50] Yan, Y. Y., Lü, Z. G. & Zheng, H. Effects of counter-rotating-wave terms of the driving field on the spectrum of resonance fluorescence. *Phys. Rev. A* **88**, 053821 (2013).

