# Supplementary material for: Drastically magnetically tuned coupling strength and nonlinearity in CrSBr exciton-polaritons
Source: Light Sci Appl. 2026 Jun 26;15:284. doi: 10.1038/s41377-026-02371-w (PMC13309558; doi:10.1038/s41377-026-02371-w)
Supplement: Supplementary file 1 — Supplementary Information [file 41377_2026_2371_MOESM1_ESM.docx]

Supplementary Information for

**Drastically Magnetically Tuned Coupling Strength and Nonlinearity in CrSBr Exciton-Polaritons**

Chun Li,^1,†^ Chao Shen,^2,3,†^ Xuekai Ma,^4^ Kwok Kwan Tang,^1^ Yutong Zhang,^5^ Nai Jiang,^2^ Xinyi Deng,^1^ Qing Wan,^1,6^ Jiepeng Song,^1^ Jiaqi Guo,^2^ Tian Lan,^1^ Hailong Fu,^7^ Feng Li,^8^ Yilin Wang,^6^ Xinfeng Liu,^5^ and Qing Zhang^1,*^

^1^School of Materials Science and Engineering, Peking University, Beijing 100871, China

^2^State Key Laboratory for Superlattices and Microstructures, Institute of Semiconductors, Chinese Academy of Sciences, Beijing 100083, China

^3^Center of Materials Science and Optoelectronics Engineering, University of Chinese Academy of Sciences, Beijing 100049, China

^4^Department of Physics and Center for Optoelectronics and Photonics Paderborn (CeOPP), Paderborn University, Warburger Strasse 100, 33098 Paderborn, Germany

^5^CAS Key Laboratory of Standardization and Measurement for Nanotechnology, CAS Center of Excellence for Nanoscience, National Center for Nanoscience and Technology, Beijing 100190, China

^6^School of Integrated Circuits, Shandong Technology Center of Nanodevices and Integration, State Key Laboratory of Crystal Materials, Shandong University, Jinan 250100, China

^7^School of Physics, Zhejiang University, Hangzhou 310058, China

^8^Key Laboratory for Physical Electronics and Devices of the Ministry of Education & Shaanxi Key Laboratory of Information Photonic Technique, School of Electronic Science and Engineering, Faculty of Electronic and Information Engineering, Xi’an Jiaotong University, Xi’an 710049, China

^*^Q_zhang@pku.edu.cn

^†^These authors contributed equally to this work.

**Supplementary Note S1. Effective refractive index dispersion**

We implement the Lorentz oscillator model to describe the optical dielectric function for X_H_ exciton along the *b*-axis, *ε*_b_, as follows^1^:

(1)

where $\varepsilon_{\text{b}}^{\infty}$ is the energy-independent background permittivity along the *b*-axis, *f* is the exciton oscillator strength, *E* is the energy, *E*_X_ is the X_H_ exciton energy (considering exciton-magnon interaction, see Note S5), ℏ is the reduced Planck constant, and *γ* is the exciton dissipative energy.

Further, the wavevector for the out-of-plane cavity mode along the *c*-axis, *k*_c_, can be expressed as:

$k_{\text{c}}=\frac{m\pi}{H}$ (2)

where *m* is the mode number, and *H* is the CrSBr crystal thickness.

For light polarized along the *b*-axis, *k*_c_ is connected with the refractive index along the *b*-axis, *n*_b_, by the equation:

(3)

where *c* is the velocity of light in a vacuum.

According to equations (1)-(3), we obtained the excellent agreement between the simulated and experimental results for all samples using *f* = 7.28 (eV)^2^ (Fig. S2) or Rabi splitting energy of 695 meV (Fig. S5).

The effective refractive index along the *a*-axis can be well fitted using the Sellmeier equation (Fig. S2).

**Supplementary Note S2. Ultrastrong coupling model for fitting exciton-polaritons**

Since the obtained normalized coupling strength using the strong coupling model exceeds 0.1, it indicates that the exciton-photon coupling may have entered the ultrastrong coupling regime. In the strong coupling regime, the exciton-polariton energy can be obtained by solving the Hamiltonian that includes only first-order effects (exciton absorption and photon emission). While in the ultrastrong coupling case, higher-order effects such as the counter-rotating terms can no longer be neglected, and the exciton-polariton energy *E* is determined by solving a biquadratic equation^2^:

$$(E^{2}-E_{\text{X}}^{2})(E^{2}-E_{\text{C}}^{2})-4gE^{2}E_{\text{C}}/E_{\text{X}}=0$$

where *E*_X_ and *E*_C_ are the exciton energy and cavity photon energy, and *g* is the coupling strength. Using the ultrastrong coupling model, we refitted the data in Fig. 1d to obtain a Rabi splitting energy of 797 meV (Fig. S9), which is slightly higher than 695 meV derived from the strong coupling model. Nevertheless, since there is no distinct critical point separating the strong and ultrastrong coupling regimes that requires a change of fitting model^3^, and we have not yet directly observed phenomena typically associated with the ultrastrong coupling, such as correlated photon pairs generation and vacuum Bloch-Siegert shift, we continue to adopt the strong coupling model in this work.

**Supplementary Note S3. Light velocity, exciton/photon fraction and effective mass of exciton-polaritons**

The light velocity can be described as *c*/*n*_b_, where *c* is the velocity of light in a vacuum and *n*_b_ is the refractive index along the *b*-axis (see Note S1).

The exciton fraction |*X*| of exciton-polaritons on the lower polariton branch can be expressed as^4^:

$\left|X\right|=\frac{1}{2}+\frac{E-E_{\text{X}}}{2\sqrt{\Omega^{2}+(E-E_{\text{X}})^{2}}}$ (1)

where *E* is the exciton-polariton energy, *E*_X_ is the X_H_ exciton energy, and Ω is the Rabi splitting energy.

The photon fraction |*C*| of exciton-polaritons on the lower polariton branch can be expressed as:

$\left|C\right|=1-\left|X\right|$ (2)

The effective mass *m*^*^ of exciton-polaritons on the lower polariton branch can be expressed as:

$\frac{1}{m^{*}}=\frac{|C|}{m_{\text{p}}}+\frac{|X|}{m_{\text{X}}}$ (3)

where *m*_p_ = 1.136 × 10^-5^*m*_0_ is the photon mass (*m*_0_ is the electron mass) and *m*_X_ = 0.20*m*_0_ is the exciton mass for CrSBr.

**Supplementary Note S4. Temperature dependence of exciton-polariton energy shift and Rabi splitting energy variation induced by changes in magnetic structure phase**

The X_H_ exciton-polariton and X_H_ exciton energies exhibit a redshift during the antiferromagnetic (AFM) to ferromagnetic (FM) phase with increased applied magnetic field due to exciton-magnon coupling, and the energy shift *E*_shift_, related to the temperature (*T*) dependence of the sublattice magnetization, follows the equation^5^:

$E_{\text{shift}}=E_{\text{shift,0}}(\frac{T_{\text{corr}}-T}{T_{\text{corr}}})^{\alpha}$ (1)

where *E*_shift,0_ is the energy shift at 0 K, *T*_corr_ is the temperature up to which magnetic field correlations are observed, and *α* is a parameter. The fitted *T*_corr_ is 139.6 K for *m* = 6 branch, 138.7 K for *m* = 5 branch, 143.3 K for *m* = 4 branch, and 141.7 K for X_H_ exciton, respectively (Fig. S16a), all of which are close to the reported Néel temperature (~132 K for bulk). The slight difference may result from the fact that short-range correlations can still survive above the magnetic structure phase transition temperature.

Similarly, the Rabi splitting energy variation ΔΩ during the transition from AFM phase to FM phase can also be described using the exciton-magnon coupling theory:

$\Delta\Omega=\Delta\Omega_{0}(\frac{T_{\text{corr}}-T}{T_{\text{corr}}})^{\alpha}$ (2)

where ΔΩ_0_ is the Rabi splitting energy variation at 0 K, *T*_corr_ is the temperature up to which magnetic field correlations are observed, and *α* is a parameter. The experimental data can be well fitted with a fitted *T*_corr_ of 140.7 K (Fig. S16b), also consistent with the Néel temperature.

**Supplementary Note S5. Temperature dependence of X_H_ exciton energy and Rabi splitting energy**

The X_H_ exciton energy *E*_X_ at a certain temperature *T* under magnetic field *B* = 0 T and *B* = 1 T can be well described by equations (1) and (2):

$E_{\text{X}}(T)=E_{\text{X}}(0)+\frac{2A_{\text{ph}1}}{\exp(\frac{E_{\text{ph}1}}{k_{\text{B}}T})-1}+\frac{2A_{\text{ph2}}}{\exp(\frac{E_{\text{ph2}}}{k_{\text{B}}T})-1}+\frac{E_{\text{shift}}}{2}$ (1)

$E_{\text{X}}(T)=E_{\text{X}}(0)+\frac{2A_{\text{ph}1}}{\exp(\frac{E_{\text{ph}1}}{k_{\text{B}}T})-1}+\frac{2A_{\text{ph2}}}{\exp(\frac{E_{\text{ph2}}}{k_{\text{B}}T})-1}-\frac{E_{\text{shift}}}{2}$ (2)

In the above equations, the first term *E*_X_(0) is the X_H_ exciton energy at 0 K. The second and third terms are due to exciton-phonon coupling, where *A*_ph1_ and *A*_ph2_ are the exciton-phonon coupling strength of the two phonons, *E*_ph1_ and *E*_ph2_ are the energy of the two phonons, and *k*_B_T is the thermal energy. The fourth term is the energy variation due to exciton-magnon coupling, as discussed in Note S4. The X_H_ exciton energy can be well fitted using the above equations (Fig. S16c), and the fitted *E*_ph1_ = 43.1 meV and *E*_ph2_ = 36.4 meV are consistent with the measured A_g_^3^ and B_2g_^3^ phonon mode energies^6^. These results confirm the rationality of the model.

Similarly, the Rabi splitting energy Ω of a certain temperature *T* under *B* = 0 T and *B* = 1 T can also be well described by the above equations after changing the exciton-phonon coupling strength *A*_ph1_ and *A*_ph2_ in equations (1) and (2) with the exciton-photon coupling coefficient *A*_EP1_ and *A*_EP2_. Fig. S16d represents the consistency between the experimental data and fitting results using *E*_ph1_ = 43.1 meV (corresponding to A_g_^3^ phonon mode) and *E*_ph2_ = 36.4 meV (corresponding to B_2g_^3^ phonon mode), indicating the exciton-photon coupling is strongly correlated with magnon and phonon.

**Supplementary Note S6. Rabi splitting energy saturation**

With increasing total excitation density *n*, the Rabi splitting energy Ω(*n*) will saturate due to Pauli blocking, which can be expressed by^7^:

$\Omega(n)=\frac{\Omega(0)}{\sqrt{1+\frac{n}{n_{\text{s}}}}}$ (1)

where Ω(0) is the Rabi splitting energy at the lowest detected excitation density, and *n*_s_ is the saturation density.

The total excitation density *n* is estimated by:

(2)

with *R* is the reflection coefficient (0.30), *A* is the absorbance (close to 1 according to the reference^8^), *A*_beam_ is the laser beam area (7 μm^2^), and *f* is the laser repetition rate (80 MHz). *P*(*ω*) and are the excitation power and excitation energy at a specific frequency, and we calculate the integration results considering all exciton-polariton frequencies.

**Supplementary Note S7. Theoretical discussion of magnon-enhanced polariton nonlinearity**

The polariton nonlinearity originates from exciton-exciton Coulomb interaction and phase-space filling^4^. The interaction strength for exciton-exciton Coulomb interaction (*g*_XX_) can be related to the exciton binding energy *E*_b_, Bohr radius *a*_B_, and reduced mass *μ* as follows^7^:

$g_{\text{XX}}\approx E_{\text{b}}a_{\text{B}}^{2}\propto\frac{1}{\mu}$ (1)

In the model of magnon-assisted exciton-exciton attraction interactions, the redshift induced by the exciton-exciton attraction interaction Δ*E*_XX_ at specific excitation density *n* depends on the angle *θ* between the magnetization vectors of the two sublattices, as expressed below^9^:

$ng_{\text{XX}}=\Delta E_{\text{XX}}=-C_{1}\cos^{2}(\frac{\theta}{2})-C_{2}\sin^{2}\theta$ (2)

where *C*_1_ (related to the redshift of the exciton energy due to changes in the magnetic order) and *C*_2_ (related to the magnon energy and redshift of the exciton energy due to changes in the magnetic order) are fitting parameters. When the magnetic field *B* is applied along the *c*-axis, the angle between the sublattice magnetizations gradually changes according to the following equation^10^:

$\cos(\frac{\theta}{2})\propto B$ (3)

By combining equations (1)-(3), one can describe the relation between *g*_XX_ (*μ*) and *B*.

The phase-space filling effect describes the reduction of available electronic and hole states as the exciton density increases. Because electrons and holes are fermions, the Pauli exclusion principle limits the number of accessible states, diminishing the exciton oscillator strength and the Rabi splitting energy. The interaction strength for the phase-space filling effect (*g*_SAT_) can be related to the exciton Bohr radius *a*_B_, and reduced mass *μ* as follows^7^.

$g_{\text{SAT}}\propto a_{\text{B}}^{2}\propto\frac{1}{\mu^{2}}$ (4)

Similar to equations (1) and (2), we can obtain:

$Ng_{\text{SAT}}=\Delta E_{\text{SAT}}\propto\left[C_{1}\cos^{2}(\frac{\theta}{2})-C_{2}\sin^{2}\theta\right]^{2}$ (5)

By combining equations (3) and (5), one can describe the relation between *g*_SAT_ and *B*.

Finally, by accounting for the excitonic fraction |*X*| and photonic fraction |*C*| of the exciton-polariton, the total energy shift Δ*E* can be described as^11^:

$\Delta E=ng_{\text{XX}}|X|^{2}+4ng_{S\text{AT}}|X|\sqrt{|X||C|}$ (6)

By combining the above equations, one can obtain the relation between *ΔE* and *B*, considering the magnon-mediated exciton-exciton interaction and phase-space filling for polariton nonlinearity.

Notably, the above analysis is based on the spin-canting (magnetic field is along the intermediate *a*-axis or hard *c*-axis). However, in our experiment, the magnetic field is applied along the easy *b*-axis, for which the relationship between the sublattice magnetization angle *θ* and the magnetic field *B* remains unclear. In such a configuration, a recent research has observed spin flipping at the intermediate magnetic (IM) phase in few-layer samples, accompanied by rapid switching between multiple magnetic states^12^. In contrast, in bulk samples, the photoluminescence redshift remains gradual, suggesting that the spin orientations in the IM state may vary continuously^13^. Besides, other reports have proposed a spin-flop mechanism using Kerr microscopy, involving both rapid flipping and gradual reorientation^14^. Another ongoing debate concerns whether non-collinear spin configurations exist in the IM state^15^. Therefore, future studies are needed to elucidate the evolution of the sublattice magnetization under a magnetic field applied along the *b-*axis, which will help in understanding the magnon-assisted exciton-polariton nonlinearity.

**Fig. S1.** Thickness dependence of differential reflectance spectra in different samples at 6 K under *B* = 0 T.

**Fig. S2.** Energy dependence of effective refractive index along the *b*-axis (navy) and *a*-axis (brown). Dots/squares: experimental data. Solid curves: fitting results.

**Fig. S3.** The differential reflectance spectra around the X_H_ exciton energy (a) and X_L_ exciton energy (b). X_H_ exciton exhibits a larger fitted amplitude A using the effective dispersive Lorentzian function^16^, suggesting a larger integrated absorption area and larger exciton oscillator strength.

**Fig. S4.** The differential reflectance spectra at 6 K of the 354 nm-thick CrSBr crystal under *B* = 0.8 T (ferromagnetic phase). The arrows indicate possible indirect optical transitions above the X_H_ exciton energy (a) and X_L_ exciton energy (b).

**Fig. S5.** Energy-wavevector dispersion of CrSBr crystals with different thicknesses. Dots: experimental data at normal incidence. Solid curves: fitting results using the coupled harmonic oscillator model, giving an average Rabi splitting energy of 695 meV. UPB (LPB) stands for the upper (lower) polariton branch.

**Fig. S6.** (a) Differential reflectance spectra as a function of polarization of reflected light at 80 K under *B* = 1 T. White dashed lines represent polarization along the *a*-axis and *b*-axis. (b) Differential reflectance spectra at 80 K under *B* = 1 T with the polarization of the reflected light along the *a*-axis (cyan) and *b*-axis (navy) and MCD spectrum at 80 K under *B* = 4 T (brown) of the 354 nm-thick CrSBr crystal.

**Fig. S7.** (a) Differential reflectance spectra as a function of polarization of reflected light at 80 K under *B* = 0 T. White dashed lines represent polarization along the *a*-axis and *b*-axis. (b) Differential reflectance spectra at 80 K under *B* = 0 T with the polarization of the reflected light along the *a*-axis (cyan) and *b*-axis (navy) of the 354 nm-thick CrSBr crystal.

**Fig. S8.** Energy-wavevector dispersion of CrSBr crystals at 6 K at five different locations with similar crystal dimensions, where the strong coupling is robust, instead of a little polariton energy and Rabi splitting energy variation.

**Fig. S9.** Energy-detuning dispersion of CrSBr crystals with different thicknesses. The detuning energy is equal to the cavity mode CM energy minus the X_H_ exciton energy. Dots: experimental data at normal incidence, same as in Fig. 1d. Navy curves: fitting results using the ultrastrong coupling model (see Note S2) with an average Rabi splitting energy of 797 meV.

**Fig. S10.** Magnetic field dependence of X_H_ exciton-polariton energies (a-d) and fitted X_H_ exciton energies (e-h) extracted from Figs. 2a (a, e), 2b (b, f), 2c (c, g), and 2d (d, h) in the main text.

**Fig. S11.** Magnetic field dependence of differential reflectance spectra (a), X_L_ exciton-polariton energies (b), fitted Rabi splitting energy (upper panel in c), and fitted X_L_ exciton energy (lower panel in c) at 6 K of the 354 nm-thick CrSBr crystal.

**Fig. S12.** (a, b) Light velocity (a) and effective mass (b) *versus* energy difference between X_H_ exciton and X_H_ exciton-polariton at 6 K under *B* = 0 T (navy) and 1 T (brown) for X_H_ exciton-polaritons. (c, d) Light velocity (c) and effective mass (d) *versus* energy difference between X_L_ exciton and X_L_ exciton-polariton at 6 K under *B* = 0 T (navy) and 1 T (brown) for X_L_ exciton-polaritons.

**Fig. S13.** Differential reflectance at 6 K under *B* = 0 T (a) and 1 T (b) of a 5 nm-thick CrSBr crystal. The open circles are experimental data, and the curves are fitting results according to the reference^7^.

**Fig. S14.** MCD intensity at 633 nm as a function of out-of-plane magnetic field at 80 K (blue), 120 K (red), and 160 K (black) for the 37 nm-thick CrSBr crystal.

**Fig. S15.** 2D colored reflectance spectra at normal incidence of the 354 nm-thick CrSBr crystal at *B* = 0 T (a) and *B* = 1 T (b). The X_H_ exciton-polariton energies are guided by white dashed curves.

**Fig. S16.** (a, b) Temperature dependence of energy shift for three X_H_ exciton-polariton branches and X_H_ exciton (a) and Rabi splitting energy variation for X_H_ exciton-polaritons (b) during the AFM-FM phase transition. The circles and stars are experimental data, and the solid curves are the fitting results. (c, d) Temperature dependence of the fitted X_H_ exciton energy (c) and Rabi splitting energy for X_H_ exciton-polaritons (d) under *B* = 0 T (navy) and 1 T (brown). The circles are experimental data and the thick curves are fitting results. The vertical dashed lines in (a-d) represent the Néel temperature.

**Fig. S17.** Representative differential reflectance spectrum at 0.13 × 10^12^ cm^-2^ under *B* = 0 T. The red curve is the fitting result using the Lorentzian function.

**Fig. S18.** Δ*E*/Δ*n* (energy shift divided by variation in excitation density) *versus* exciton fraction extracted from Fig. 3b, giving a fitted 4*g*_SAT_ = 0.72 μeV μm^2^, quite larger than *g*_XX_ = -0.10 μeV μm^2^, demonstrating the dominance of phase-space filling in polariton nonlinearity. Blue dots are experimental data, the black solid curve is the fitting result according to equation (6) in Note S7, and the blue (red) dashed curve is the contribution of phase-space filling effect (exciton-exciton interaction).

**References**

1. A. Kavokin, *Microcavities*, OUP Oxford, (2011).

2. D. G. Baranov, B. Munkhbat, E. Zhukova, et al., Ultrastrong coupling between nanoparticle plasmons and cavity photons at ambient conditions, *Nat. Commun.* **11**, 2715 (2020).

3. S. B. Anantharaman, J. Lynch, M. Aleksich, et al., Ultrastrong light–matter coupling in two-dimensional metal–organic chalcogenolates, *Nat. Photonics* **19**, 322 (2025).

4. H. Deng, H. Haug, Y. Yamamoto, Exciton-polariton Bose-Einstein condensation, *Rev. Mod. Phys.* **82**, 1489 (2010).

5. F. Dirnberger, J. Quan, R. Bushati, et al., Magneto-optics in a van der Waals magnet tuned by self-hybridized polaritons, *Nature* **620**, 533 (2023).

6. A. Pawbake, T. Pelini, N. P. Wilson, et al., Raman scattering signatures of strong spin-phonon coupling in the bulk magnetic van der Waals material CrSBr, *Phys. Rev. B* **107**, 075421 (2023).

7. L. Zhang, F. Wu, S. Hou, et al., Van der Waals heterostructure polaritons with moiré-induced nonlinearity, *Nature* **591**, 61 (2021).

8. Q. Li, X. Xie, A. Alfrey, et al., Two-Dimensional Magnetic Exciton Polariton with Strongly Coupled Atomic and Photonic Anisotropies, *Phys. Rev. Lett.* **133**, 266901 (2024).

9. B. Datta, P. C. Adak, S. Yu, et al., Magnon-mediated exciton-exciton interaction in a van der Waals antiferromagnet, *Nat. Mater.* **24**, 1027 (2025).

10. N. P. Wilson, K. Lee, J. Cenker, et al., Interlayer electronic coupling on demand in a 2D magnetic semiconductor, *Nat. Mater.* **20**, 1657 (2020).

11. F. Barachati, A. Fieramosca, S. Hafezian, et al., Interacting polariton fluids in a monolayer of tungsten disulfide, *Nat. Nanotechnol.* **13**, 906 (2018).

12. Z. Sun, C. Hong, Y. Chen, et al., Resolving and routing magnetic polymorphs in a 2D layered antiferromagnet, *Nat. Mater.* **24**, 226 (2025).

13. C. Li, C. Shen, N. Jiang, et al., 2D CrSBr Enables Magnetically Controllable Exciton-Polaritons in an Open Cavity, *Adv. Funct. Mater.* **34**, 2411589 (2024).

14. J. Yu, D. Liu, Z. Ding, et al., Direct Imaging of Antiferromagnet-Ferromagnet Phase Transition in van der Waals Antiferromagnet CrSBr, *Adv. Funct. Mater.* **34**, 2307259 (2024).

15. C. Ye, C. Wang, Q. Wu, et al., Layer-Dependent Interlayer Antiferromagnetic Spin Reorientation in Air-Stable Semiconductor CrSBr, *ACS Nano* **16**, 11876 (2022).

16. T. Smoleński, P. E. Dolgirev, C. Kuhlenkamp, et al., Signatures of Wigner crystal of electrons in a monolayer semiconductor, *Nature* **595**, 53 (2021).
